# Trends in Prenatal Substance Use Across Ontario, Canada

**DOI:** 10.1001/jamanetworkopen.2024.55310

**Published:** 2025-01-21

**Authors:** Gabrielle Pratt Tremblay, Sheryll Dimanlig-Cruz, Anna Dion, Daniel J. Corsi

**Affiliations:** 1 School of Epidemiology and Public Health, Faculty of Medicine, University of Ottawa, Ottawa, Ontario, Canada; 2 Better Outcomes Registry and Network Ontario, Ottawa, Ontario, Canada; 3 CHEO Research Institute, Ottawa, Ontario, Canada

## Abstract

This cross-sectional study investigates trends in use of cannabis, tobacco, and alcohol among pregnant individuals in Ontario, Canada, from 2012 to 2022.

## Introduction

Tobacco, alcohol, and cannabis are consumed in up to 1 in 10 pregnancies.^1^ Prenatal substance use is associated with several adverse pregnancy, neonatal, postneonatal, and long-term outcomes, including increased risk of miscarriage, stillbirth, preterm birth, small size at birth, and neurodevelopmental outcomes.^2,3^ The extent to which substance use behaviors have evolved in recent years by substance type and maternal age is not well described. We used data from Ontario, Canada, to examine trends in the prenatal prevalence of cannabis, tobacco, and alcohol use between 2012 and 2022.

## Methods

The Children’s Hospital of Eastern Ontario Research Ethics Board approved this cross-sectional study, which adhered to the STROBE reporting guideline. Consent was waived for the Better Outcomes Registry and Network (BORN) under the Personal Health Information Protection Act 2004. Maternal-newborn records of deliveries between April 1, 2012, and March 31, 2022, were obtained from BORN. We included Ontario residents aged 12 to 54 years with singleton deliveries, yielding an analytic sample of 975 242 individuals (eMethods in Supplement 1). Prenatal cannabis, alcohol, and tobacco use were collected via patient disclosure during prenatal care and from clinical records. We calculated annual substance use prevalence overall and by age and compared these over time. Statistical analyses used 2-sided tests with an α of .05 and were performed using SAS statistical software version 9.4 (SAS Institute).

## Results

**Figure 1.**
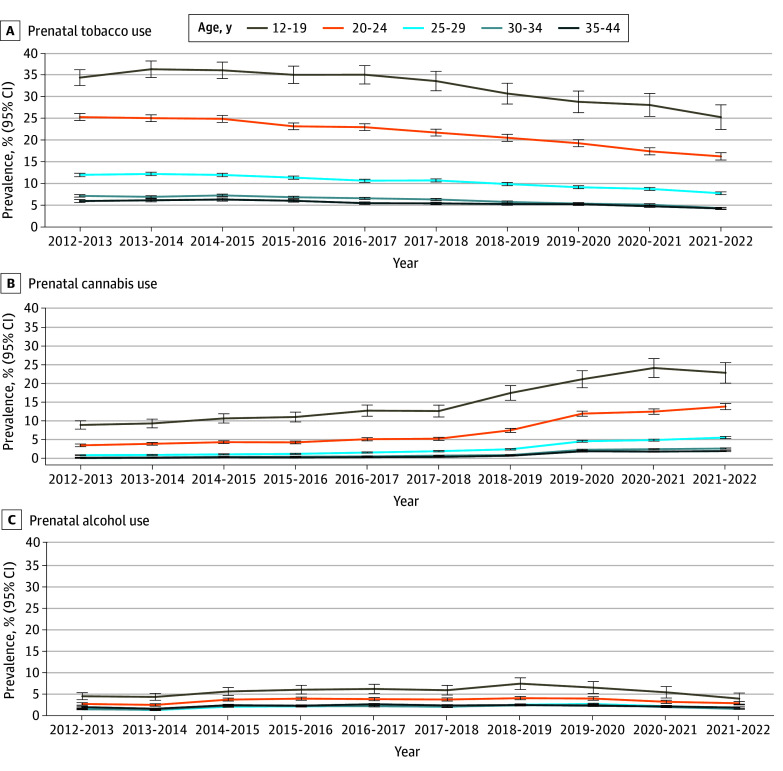
Trends in Prevalence of Prenatal Substance Use Whiskers indicate 95% CIs.

Of 975 242 pregnant women (385 463 aged <30 years [39.5%]; mean [SD] maternal age at birth, 30.7 [5.2] years), 119 702 individuals (12.3%) consumed at least 1 substance during pregnancy. Overall, 23 372 individuals (2.4%) used cannabis, 89 968 individuals (9.2%) smoked tobacco, and 23 331 individuals (2.4%) consumed alcohol in pregnancy. Between 2012 and 2022, we observed a 45.7% decrease in prenatal tobacco use across all age groups, from 11.3% (95% CI, 11.1%-11.5%) in 2012 to 6.1% (95% CI, 6.0%-6.3%) in 2022 (*P* < .001) (Figure 1A). Tobacco use was consistently higher among mothers aged 12 to 19 years, and use decreased with increasing age. Despite decreases, smoking in pregnancy remained high among young mothers in 2022, with 25.2% (95% CI, 22.3%-28.0%) of women aged 12 to 19 years and 16.2% (95% CI, 15.3%-12.0%) of women aged 20 to 24 years using tobacco.

**Figure 2.**
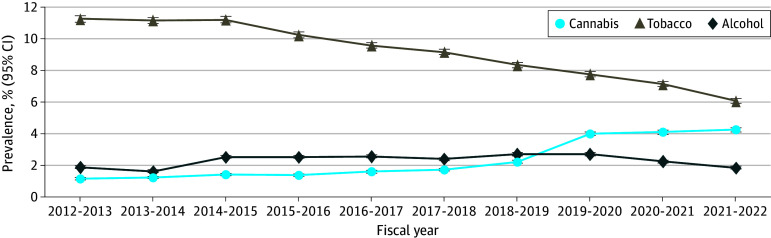
Year-to-Year Prevalence of Prenatal Substance Use The study population includes 2342 mothers (0.2%) aged 44 to 55 years. Whiskers indicate 95% CIs.

Unlike tobacco, cannabis use increased from 1.2% (95% CI, 1.2%-1.3%) in 2012 to 4.3% (95% CI, 4.2%-4.5%) in 2022 (Figure 1B). Like tobacco, cannabis was more frequently consumed by younger women, reaching a prevalence of 24.1% (95% CI, 21.6%-26.6%) among those aged 12 to 19 years in 2020-2021 before declining to 22.9% (95% CI, 20.1%-25.6%) in 2021-2022. After 2018-2019, we observed a marked increase in cannabis consumption across all age groups (Figure 2).

Alcohol consumption among pregnant women remained relatively stable across age groups (Figure 1C), with year-to-year fluctuations. Prenatal alcohol consumption was highest among women aged 12 to 19 years.

## Discussion

In this cross-sectional study, we observed a nearly 50% decrease in prenatal tobacco consumption and a 2.5-fold increase in the prevalence of prenatal cannabis use across all ages between 2012 and 2022, with alcohol use remaining stable. Tobacco consumption decreased, similar to US trends,^4^ but remained high among mothers younger than 25 years. Declining smoking levels and low levels of alcohol use may, in part, be associated with interventions targeting cessation and prevention of alcohol and tobacco use in pregnancy, although these may need further targeting to younger or first-time mothers. In contrast, the recent legalization of cannabis, coupled with increasing social acceptability, may foster a lower perceived risk for cannabis use and increase individuals’ willingness to report.^5^ In addition, the COVID-19 pandemic led to increased substance use in specific demographics, including pregnant individuals.^6^ This study’s limitations include the use of a self-reported exposure variable, which likely underreported substance use. Future research should prioritize identifying underlying factors driving trends in prenatal substance use as part of strategies to improve maternal and child health.

## Notes

Supplement 1.
eMethods.

eReferences.

Supplement 2.
Data Sharing Statement

## References

[1] Public Health Ontario. Maternal Health Snapshot: PHU (2013 to 2021). Accessed September 15, 2024. https://www.publichealthontario.ca/en/Data-and-Analysis/Reproductive-and-Child-Health/Maternal-Health

[2] Corsi DJ, Donelle J, Sucha E, . Maternal cannabis use in pregnancy and child neurodevelopmental outcomes. Nat Med. 2020;26(10):1536-1540. doi:10.1038/s41591-020-1002-5 32778828

[3] Prince MK, Daley SF, Ayers D. Substance use in pregnancy. In: StatPearls. StatPearls Publishing; 2024. 31194470

[4] Zaganjor I, Kramer RD, Kofie JN, Sawdey MD, Cullen KA. Trends in smoking before, during, and after pregnancy in the United States from 2000 to 2020: Pregnancy Risk Assessment Monitoring System. J Womens Health (Larchmt). 2024;33(3):283-293. doi:10.1089/jwh.2023.0641 38153374

[5] Azofeifa A, Mattson ME, Schauer G, McAfee T, Grant A, Lyerla R. National estimates of marijuana use and related indicators—National Survey on Drug Use and Health, United States, 2002-2014. MMWR Surveill Summ. 2016;65(11):1-28. doi:10.15585/mmwr.ss6511a1 27584586

[6] Frank O, Murphy MSQ, Talarico R, . The COVID-19 pandemic and parental substance use: a cross-sectional survey of substance use among pregnant and post-partum individuals and their partners. J Subst Use. 2023;29(3):477-485. doi:10.1080/14659891.2023.2183148

