# Supplementary material for: Trends in Prenatal Substance Use Across Ontario, Canada
Source: JAMA Netw Open. 2025 Jan 21;8(1):e2455310. doi: 10.1001/jamanetworkopen.2024.55310 (PMC11751739; doi:10.1001/jamanetworkopen.2024.55310)
Supplement: Supplement 1. — eMethods. eReferences. [file jamanetwopen-e2455310-s001.pdf]

Supplemental Online Content

Pratt Tremblay G, Dimanlig-Cruz S, Dion A, Corsi DJ. Trends in prenatal substance use across Ontario, Canada. *JAMA Netw Open*. 2025;8(1):e2455310. doi:10.1001/jamanetworkopen.2024.55310

eMethods.
eReferences.

This supplemental material has been provided by the authors to give readers additional information about their work.

© 2025 Pratt Tremblay G et al. *JAMA Network Open*.

eMethods.

Research ethics board approval for this study was obtained from the Children's Hospital of Eastern Ontario Research Ethics Board. Under the Personal Health Information Protection Act, 2004, Ontario's Better Outcomes Registry & Network (BORN) can collect and use personal health information without consent for facilitating or improving healthcare.

Study population and data source

The Better Outcomes Registry & Network (BORN) Ontario (https://www.bornontario.ca) is a perinatal registry in Ontario that, under provincial legislation, can collect and use personal health information to facilitate and improve health care. BORN captures nearly all births occurring in Ontario, about 40% of annual births in Canada.[1] Data collected in BORN include demographic information, obstetric history, health behaviours and substance use, pre-existing conditions, obstetric complications, and birth outcomes. Data are collected from perinatal records, clinical forms, and patient interviews during admission for labour and delivery. Specifically, for substance use, data are collected via the Ontario Perinatal Record and typically gathered at the first prenatal consult, occurring around the 11th week of gestation.[2]

Previous validation studies and Data quality audits of BORN data have indicated high levels of completeness (less than 10% missing) and high levels of accuracy (85% with Kappa>0.6) in selected fields, including maternal tobacco use.[1] We have also previously conducted validation studies on substance use data. We found that, for example, cannabis use defined in BORN had a sensitivity of 97% (95% CI: 93-99) and a specificity of 94% (91-96) compared to clinical records, and the positive predictive value was 90% (85-94).[3]

In the current study, we conducted a retrospective cohort analysis including Ontario residents at least 12 years of age who delivered a singleton infant at a gestational age ≥ 20 weeks

© 2025 Pratt Tremblay G et al. *JAMA Network Open*.

in an Ontario hospital between April 1, 2012, and March 31, 2022. A total of 1,427,154 records were obtained from hospital deliveries. We excluded infants below 200g and records with missing data on substance use exposures and other covariates, including maternal age, parity, pre-existing health conditions, and socioeconomic characteristics that will be used in subsequent analyses (n=451,912), yielding an analytic sample of yielding an analytic sample of 975,242.

Substance Use in Pregnancy

Maternal use of alcohol, cannabis, and tobacco was recorded during routine prenatal care for mothers, as previously described.[2,3] A standardized perinatal record is completed for all pregnant women with their obstetrician, family physician or midwife. At the first prenatal visit, women are explicitly asked about substance use in pregnancy. The question is recorded as 'yes, use of cannabis' or 'no' for the current pregnancy, and similarly for tobacco and alcohol. Data on the frequency, trimester, and duration of substance use are limited and not considered in the present study. Data from the perinatal record are then abstracted into the BORN registry. In addition, substance use can also be captured from clinical histories obtained from patients at admission to the hospital for labour and delivery and updated into the BORN record.

Statistical analysis

We analyzed the data between March 2023 and September 2024 using SAS version 9.4. Annual prevalence and binomial 95% confidence intervals were calculated for 1) prenatal cannabis use, 2) prenatal tobacco use, and 3) prenatal alcohol use overall and by age category from 2012-2022. Trends were assessed using a 2-sided Cochran-Armitage Test, with an alpha of 0.05. The relative % changes in prevalence were calculated as: $Prevalence_{2022}$ – $Prevalence_{2012}$ / $Prevalence_{2012}$ x 100.

Efforts to Avoid Potential Bias

© 2025 Pratt Tremblay G et al. *JAMA Network Open*.

In a cross-sectional study, biases of concern include selection bias, information bias and self-selection bias. In this study, selection bias and self-selection bias are limited as all mothers who delivered a child in a hospital between April 1, 2012, and March 2022, in Ontario, Canada, not meeting any exclusion criteria were included. All ages were assessed consistently, and mothers did not choose whether they participated in the study or not.

Information bias was stated as a limitation in this study. Prenatal substance use (exposure) was self-reported and is subject to recall bias and social desirability bias, which could have led to misclassification. Individuals might have been more hesitant to admit their substance use due to fear of the consequences and stigmatization[4,5], particularly before the legalization of cannabis in 2018 or have forgotten if they consumed it in early pregnancy. The effects of underreporting or misclassification would likely result in conservative estimates of substance use prevalence.

© 2025 Pratt Tremblay G et al. *JAMA Network Open*.

eReferences.

1. Dunn S, Bottomley J, Ali A, Walker M. 2008 Niday Perinatal Database quality audit: report of a quality assurance project. *Chronic Dis Inj Can*. Dec 2011;32(1):32-42.

2. Corsi DJ, Donelle J, Sucha E, et al. Maternal cannabis use in pregnancy and child neurodevelopmental outcomes. *Nature Medicine*. 2020/10/01 2020;26(10):1536-1540. doi:10.1038/s41591-020-1002-5

3. Corsi DJ, Walsh L, Weiss D, et al. Association Between Self-reported Prenatal Cannabis Use and Maternal, Perinatal, and Neonatal Outcomes. *JAMA*. 2019;322(2):145-152. doi:10.1001/jama.2019.8734

4. Stone R. Pregnant women and substance use: fear, stigma, and barriers to care. *Health & Justice*. 2015;3(1):2.

5. Johnson T, Fendrich M. Modeling sources of self-report bias in a survey of drug use epidemiology. *Ann Epidemiol*. May 2005;15(5):381-9. doi:10.1016/j.annepidem.2004.09.004

© 2025 Pratt Tremblay G et al. *JAMA Network Open*.
