# Supplementary material for: Trends in Prenatal Substance Use Across Ontario, Canada
Source: JAMA Netw Open. 2025 Jan 21;8(1):e2455310. doi: 10.1001/jamanetworkopen.2024.55310 (PMC11751739; doi:10.1001/jamanetworkopen.2024.55310)
Supplement: Supplement 2. — Data Sharing Statement [file jamanetwopen-e2455310-s002.pdf]

Data Sharing Statement

Pratt Tremblay. Trends in Prenatal Substance Use Across Ontario, Canada. *JAMA Netw Open*. Published online January 21, 2025. doi:10.1001/jamanetworkopen.2024.55310

Data
Data available: No

Additional Information
Explanation for why data not available: Aggregate data can be made available; however individual data requests must be made through BORN Ontario following research review.
